# Comment on Liberski, Gajos, Sikorska, and Lindenbaum: “Kuru, the First Human Prion Disease” *Viruses* 2019, 11, 232

**DOI:** 10.3390/v12030284

**Published:** 2020-03-05

**Authors:** William Arens

**Affiliations:** College of Arts and Sciences, Stony Brook University, Stony Brook, NY 11794, USA

I would like to comment on an unsupported charge published in *Viruses* [1].

“William Arens, an anthropologist, said that cannibalism as an accepted social custom did not exist, a view that was finding a receptive public audience, but one that is discredited today (52)”. The note (52) cites my book, *The Man-Eating Myth: Anthropology and Anthropophagy* [2].

I did not write that cannibalism as an accepted social custom did not exist. In fact, I argued much the opposite. To quote from the book: 

“…the question of whether or not people eat each other is taken as interesting but moot. But if the idea is commonly accepted without adequate documentation, then the reason for this state of affairs is an even more intriguing problem…” [2].

Some of my anthropological colleagues grasped this argument easily. A review in the *TLS* [3] for example, states that “Arens does not deny” the “possibility that there really are, or have been, man-eaters somewhere in the world…”. Another reviewer in *American Ethnologist* [4] states “Arens does not claim to prove that cannibalism never existed as a custom (which clearly would be impossible), but he has raised convincing doubts that it ever did.”

Nor is my argument beyond the comprehension of the anonymous authors of the Wikipedia entry: “(Arens) nevertheless refused to rule out the possibility that (cannibalism) had ever occurred, maintaining that the correct methodological stance was to hold an open mind on the issue....” [5].

I admit that *The Man-Eating Myth* earned mixed reviews from my colleagues. A significant contention was its implication that some of my anthropological colleagues had promoted cultural misunderstanding by demonizing “the other,” in what amounted to a racial slur on indigenous people. This portrait may not have been their intention, but it would be disingenuous to deny this outcome for the lay public.

Far from being “discredited” the *Man-Eating Myth* was chosen as the focal text of the Annual Essex Symposium in the UK. Its argument was then adopted as the theme for the resulting volume *Cannibalism and the Colonial World* [6]. Later, the book was accorded the same treatment at a similar gathering at Uppsala University, Sweden, where I was awarded an Honorary Doctorate from Goteborg University for advancing intercultural understanding, as well as a Rockefeller Fellowship to support work on this specific thesis. Subsequent outlets for my thesis followed. 

Finally, *The Man-Eating Myth* is still in print, and has sold over 10,000 copies in English alone. It has been translated into Italian, Spanish, Polish, Japanese, and–this year—Chinese. These results are not the common fate of a discredited book or author. 

Initially, perhaps I was overly hard on those of my anthropological colleagues who took to heart native elders’ stories that I understood, then and now, to be fairy tales. In my defense, I can only say that I was definitely young and—just possibly—rash. 
